# Deciphering genotype and geography dependent microbiome composition and its role in disease suppression in Ashwagandha

**DOI:** 10.3389/fmicb.2026.1786817

**Published:** 2026-03-20

**Authors:** Samadhan Yuvraj Bagul, Shreedevasena S, Parmeshwar Lal Saran, Ganesh Navnath Khadke, Manish Das

**Affiliations:** ICAR-Directorate of Medicinal and Aromatic Plants Research, Boriavi, Anand, India

**Keywords:** Ashwagandha, landrace, microbiome, Nagori, rhizosphere

## Abstract

Ashwagandha, *Withania somnifera* (L.) Dunal is a perennial evergreen shrub widely used to treat mental health disorders and physical debility, and to enhance overall physiological function. Variations in genotype and geographic origin significantly influence rhizospheric microbial communities by altering soil physicochemical properties. This study applied shotgun metagenomic sequencing to investigate microbial community shifts in the rhizosphere of Nagori Ashwagandha (RN) from Rajasthan, Vallabh Ashwagandha-1 (GV) from Gujarat, and Nagori Ashwagandha from Rajasthan cultivated in Gujarat (GN). Fusarium wilt incidence was 67%, affecting the roots, which represent the most economically important part of ashwagandha. Taxonomic analysis identified Actinomycetota (46–60%) and Pseudomonadota (35–42%) as the predominant phyla, with *Nocardioides* (3.1–8.8%), *Streptomyces* (4.5–6.5%), and *Bradyrhizobium* (1–1.6%) as dominant genera across all groups in metagenomic analysis. Alpha-diversity analysis revealed higher species richness and Simpson's index in the GV group compared to the GN and RN groups. Beta-diversity assessment using Bray–Curtis distances showed partial clustering of GN and RN relative to GV in principal coordinate analysis and hierarchical dendrograms. Functional profiling based on KEGG annotation indicated that core metabolic and cellular pathways predominated across all genotypes, with no significant differences in Tier 1 and Tier 2 functional categories. To our knowledge, this represents the first shotgun metagenomic analysis of ashwagandha. Culturomics analysis yielded seventeen isolates from two rhizospheric locations; among these, *Bacillus subtilis* DMA1 exhibited the highest mycelial inhibition against *Fusarium solani* (64%), with a germination rate of 98%, root length of 2.1 cm, shoot length of 1.3 cm, seed vigor index of 333.2, and maximum fresh biomass of 1.12 g. Co-inoculation with *F. solani* and *Bacillus subtilis* DMA1 in pot trials significantly increased root length (20.1 cm), shoot length (39.5 cm), root girth (14.9 mm), and total biomass (51.1 g) compared to control and *Fusarium*-only treatments. These findings indicate that *Bacillus subtilis* DMA1 reduced wilt incidence by 70% and enhanced plant growth under pathogen-stress conditions.

## Graphical Abstract

**Figure:**
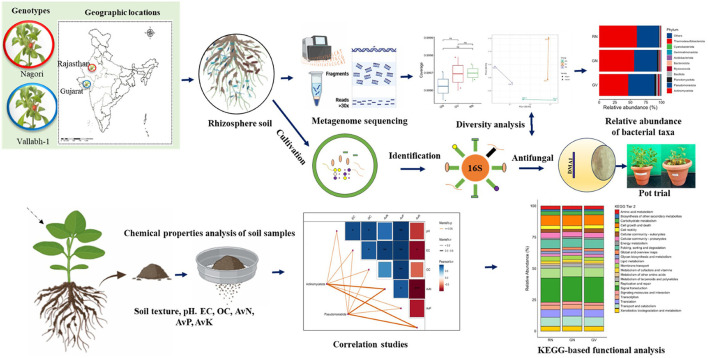

## 1 Introduction

Ashwagandha (*Withania somnifera* (L) Dunal) is a dry, woody herb widely cultivated in tropical and subtropical regions of Asia, Africa, and parts of Europe (Paul et al., 2021). The species exhibits multiple morphotypes characterized by variations in berry color, root thickness, root brittleness, and a harvesting cycle of 180 to 300 days (Saran and Das, 2023; Saran et al., 2025a). Recently, the landrace ‘Nagori Ashwagandha' was officially registered under the Geographical Indications (GI) system as “The Fringes of the ‘Thar Desert' and ‘Nagori' Ashwagandha” by the Nagauri Welfare Society, based in Nagaur, Rajasthan (GI Application No. 1143; accessed on 19.02.2026), highlighting its importance within the Indian medicinal system. Ashwagandha, a powerful adaptogenic medicinal herb used in Ayurvedic medicine, is widely used to control chronic stress and anxiety, treat insomnia, boost immune function, enhance memory and cognitive function, and exhibit strong anti-inflammatory and antioxidant properties. It also plays a role in boosting testosterone levels and improving fertility in men. It lowers blood sugar and insulin resistance, modulates thyroid hormone levels, reduces blood pressure and cholesterol, and supports cardiovascular health (Mikulska et al., 2023; Sprengel et al., 2025). Different parts of the plant, such as the roots, shoots, leaves, and fruits, are generally used to prepare herbal medicines. However, the root extract of Ashwagandha is more commonly used in the production of commercial herbal supplements (Sengupta et al., 2018; Shinde et al., 2023; Saran et al., 2025b). India is the largest exporter of ashwagandha, producing an average of 6,720.7 metric tons of dry root from 10,000 hectares of cultivated land (Pandey et al., 2024). Over the past decade, India exported 26,784 shipments of ashwagandha through 1,683 exporters to 6,419 international buyers, highlighting its dominant role in the global ashwagandha market (Berra et al., 2024). The term “rhizospheric microbiome” refers to the community of microorganisms residing in the root zone of plants (Li et al., 2021; Ling et al., 2022). Different plant varieties secrete distinct root exudates, which strongly influence the structure and composition of microbial communities in the rhizosphere, phyllosphere, and endosphere. The rhizospheric microbiome is a highly engineered microbial community that is essential for several processes associated with plant nutrient uptake, plant growth promotion, and secondary metabolite biosynthesis, their contributions to plant through pathogen suppression, competition for niches, and induction of host immunity is well documented (Kushwaha et al., 2021; El sayed et al., 2023). Plants emit ‘cry for help' signals through their associated microbiome, which leads to the selective recruitment of beneficial microbes from the soil. This microbiome-mediated protective effect, often referred to as a soil-borne legacy, can persist across subsequent plant generations (Courtney et al., 2026). Plant genotype governs immune signaling, defense responses, and the production of secondary metabolites. The colonization of specific microbes depends on host-specific recognition. Deeper root architecture promotes more lateral roots, which provide ideal microhabitats for colonization by diverse microbes. Several environmental factors, such as soil physical, chemical, and biological properties; climatic conditions, including temperature and humidity; and plant genotypes, greatly influence the composition and function of the rhizosphere microbiota (Zhang et al., 2021; Park et al., 2023). Therefore, it is essential to investigate how different genotypes interact with soil properties and shape the surrounding microbial communities. These understandings of interactions promote microbiome-assisted breeding that leverages diverse beneficial microbiomes to enhance tolerance to abiotic and biotic stresses, improve nutrient use efficiency, and increase metabolite accumulation in medicinal plants. Previous research on medicinal plants has often relied on amplicon-based sequencing approaches (Mishra et al., 2021; Steinberger et al., 2024; Wang et al., 2023; Zhang et al., 2025). However, very few studies have investigated the rhizosphere microbiome of *Withania somnifera* root microbiome using either amplicon or shotgun metagenomic approaches. Amplicon sequencing is widely used for microbial taxonomic profiling and functional prediction due to its cost-effectiveness and relatively simple data analysis (Bharti and Grimm, 2021). It has been applied successfully in various environments, including soil (Oliveira et al., 2022; He et al., 2023), water (Galagoda et al., 2023), and animal gut systems (Thakur et al., 2022). However, this approach has limitations, including primer bias, restricted taxonomic resolution (often limited to the genus level), and limited insight into microbial functional capacity. To overcome these limitations, shotgun metagenomic sequencing provides a more detailed view of microbial communities by allowing strain-level identification and direct access to the functional gene content of the microbiome (Bharti and Grimm, 2021). This includes information on metabolic pathways, resistance genes, and other important microbial functions. These data are particularly useful for exploring complex plant–microbe interactions and their role in influencing plant health and secondary metabolite production (Li et al., 2024; Wiseschart et al., 2019). Thakur et al. (2025) employed shotgun sequencing to investigate microbial responses to tillage and crop-residue management, emphasizing their significance for nutrient cycling. A study by Yue et al. (2024) found evidence that microbiome assembly depends on genotype and showed the potential for using host-driven microbial recruitment, especially key fungi like *Mortierella*, in the wheat rhizosphere

Microbiome profiling of medicinal plants, such as Ashwagandha, elucidates how root-associated microbes affect secondary metabolite accumulation and plant adaptation to environmental stresses. The present study characterizes and compares the rhizospheric microbiomes of two widely cultivated Ashwagandha varieties, Nagori and Vallabh Ashwagandha-1, through shotgun metagenomic sequencing. Although Ashwagandha is recognized for its medicinal properties, the role of rhizosphere microbes in disease suppression remains underexplored. This study analyzes the rhizosphere microbiome in relation to the ashwagandha wilt pathogen *Fusarium solani*, a persistent challenge. Examining genotype-specific rhizosphere microbiomes enables the identification of microbial consortia associated with pathogen suppression and assesses how plant genotype influences the composition and function of the rhizosphere microbiome, particularly with respect to disease-suppressive communities. The findings aim to inform the development of microbiome-assisted strategies for sustainable wilt management and enhanced therapeutic quality in Ashwagandha.

## 2 Materials and methods

### 2.1 Sample collection and experimental setup

The study was conducted at two locations in India, and details of the experimental sites are provided in Table 1. Vallabh Ashwagandha-1 and Landrace Nagori Ashwagandha were selected for comparative microbiome compositional analysis and cultivated in both Gujarat and Rajasthan. Rhizosphere soil samples were collected from five randomly selected plants per plot at maturity (180 days). Soil adhering to the root surface was brushed off and pooled to form a single composite rhizosphere sample. Sampling was conducted at two independent locations, with two biological replicates collected at each location. These replicates were divided into two fractions for metagenomic and culturomics analyses of microbial diversity. Root samples exhibiting symptoms of *Fusarium* infection in Ashwagandha were collected, and disease incidence was assessed and recorded at both study sites.

**Table 1:** Brief description of the sites and experimental design.

**Group**	**Sample name**	**Seed variety**	**Soil type**	**Exact site**	**Lat/Lon**
GN	GN-1	Landrace ‘Nagori Ashwgandha'	Clay loam	Boriavi, Anand, Gujarat, India	22°59′94” N; 72°93′36” E
	GN-2				
GV	GV-1	Vallabh Ashwagandha-1	Clay loam	Lambhvel, Anand, Gujarat, India	23°34′07” N; 72°56′38” E
	GV-2				
RN	RN-1	Landrace ‘Nagori Ashwgandha'	Sandy loam type	Churu, Rajasthan, India	27°40′31” N, 74°08′55.3” E
	RN-2				

GN, Nagori Ashwagandha grown in Anand; GV, Vallabh Ahwagandha-1 grown in Gujarat; RN, Nagori Ashwgandha grown in Rajasthan.

### 2.2 Metagenomic library preparation, diversity, analysis, and functional analysis

Metagenomic DNA was extracted from rhizospheric soils using the PowerSoil DNA extraction kit (Qiagen, USA). DNA quality was verified by gel electrophoresis, and concentration was measured with a Nanodrop spectrophotometer (Thermo Fisher Scientific, USA). Sequencing libraries were prepared using the Illumina Nextera XT DNA Library Preparation Kit (Illumina, Inc., USA). Library insert selection was performed with an Agilent TapeStation 4150 system and high-sensitivity D1000 screentape (Agilent, USA). The V3-V4 hypervariable regions of the 16S rRNA gene were amplified using region-specific primers (Forward primer: CCTAYGGGRBGCASCAG; Reverse primer: GGACTACNNGGGTATCTAAT) (Hjelmsø et al., 2014). Shotgun metagenomic sequencing was conducted on an Illumina NovaSeq 6000 system using 2 × 150 bp paired-end chemistry at Unigenome Pvt. Ltd, Ahmedabad, India. Quality assessment of raw reads was performed using FastQC v.0.11.9 with default parameters (Andrews, 2017). Adapter trimming was conducted using Fastp v.0.20.1 (parameters: –trim_front1 2 –trim_front2 2 –trim_tail1 0 –trim_tail2 0 –length_required 50 –correction –trim_poly_g –qualified_quality_phred 30) (Chen et al., 2018), followed by a subsequent quality re-assessment with FastQC (Andrews, 2017). High-quality reads were annotated using Kraken2 v.2.1.1 (Wood et al., 2019) against the RefSeq standard database. Taxonomic abundances were analyzed using the Bracken Bayesian method (Lu et al., 2017) with default parameters. Bracken outputs were converted into a biological observation matrix (BIOM) using the kraken-biom tool (Dabdoub, 2016). Amplicon sequence variants were generated, filtered, and taxonomically classified using a Naive Bayes classifier trained on the SILVA rRNA gene database (Release 138) for the extracted amplicon region. Classification was implemented in QIIME2 using the feature-classifier plugin (Bokulich et al., 2018). Relative abundances were extracted from BIOM files and summarized using the summarize_taxa.py script within the QIIME v.1.9 package (Caporaso et al., 2010). Metagenomic sequences are deposited in the NCBI SRA under biosample accession (SAMN56297678, SAMN56297679, SAMN56297680).

### 2.3 Assessment of chemical properties of the soil

Soil samples collected from the experimental site were analyzed for pH and electrical conductivity (EC) following the protocol described by Jackson (1973). Soil organic carbon (SOC) was determined using the Walkley and Black (1934) wet oxidation method. Available nitrogen (AvN) was quantified using the alkaline permanganate method (Subbiah and Asija, 1956). The ascorbic acid reductant method was employed to evaluate available phosphorus (AvP) content (Olsen, 1954). Available potassium (AvK) content was measured by flame photometry using a neutral 1N ammonium acetate (NH_4_CH_3_CO_2_) extractant (Jackson, 1973).

### 2.4 Identification of native bacterial isolates from Ashwagandha rhizosphere

Rhizosphere soil samples from both locations were serially diluted to 10^−6^ and 10^−7^, and 100 μl from each dilution was spread onto Nutrient Agar, ISP medium-5, and ISP medium-2. Plates were incubated at 28 ±2 °C for 3–7 days. Bacterial colonies were subcultured onto fresh media to obtain pure cultures. Genomic DNA was extracted from purified bacterial isolates using the QIAprep spin Miniprep kit according to the manufacturer's protocol. DNA quality and concentration were assessed using a Denovix spectrophotometer (USA), and integrity was confirmed on a 1% agarose gel. Bacterial DNA was amplified using universal primers (27F: 5′ AGAGTTTGATCMTGGCTCAG-3′, 1492R: 5′-TACGGYTACCTTGTTACGACTT-3′). PCR reactions were conducted in a 20 μL volume containing 1 μL template DNA, 0.5 μM of each primer, 10 μL of 2 × PCR master mix, and nuclease-free water. Amplification conditions consisted of an initial denaturation at 94 °C for 5 min, followed by 35 cycles of 94 °C for 30 s, 55 °C for 45 s, and 72 °C for 1 min, with a final extension at 72 °C for 7 min. Amplicons were visualized on a 1.5% agarose gel stained with ethidium bromide and submitted for sequencing via commercial services. Raw sequences were edited and aligned using MEGA 11. Taxonomic identity was determined by comparing sequences against the NCBI GenBank database using BLASTn.

### 2.5 *In vitro* antifungal assay against Ashwagandha wilt

The antifungal activity of cultured rhizosphere soil isolates against *Fusarium solani* was assessed using a dual-culture assay on potato dextrose agar (PDA). A 5 mm mycelial disc of *Fusarium solani* was placed on one side of the PDA plate, and the rhizospheric isolate was streaked on the opposite side. Plates were incubated at 28 ± 2 °C for 5 to 7 days, with pathogen-only plates serving as controls. Experiments were conducted in triplicate. Antagonistic activity was quantified by measuring the pathogen's radial growth inhibition. The percentage inhibition of mycelial growth (PIMG) was calculated as follows: PIMG (%) = [(R1 - R_2_)/R1] × 100, where R1 represents radial growth in the control plate, and R_2_ represents growth in the dual culture plate. Isolates exhibiting a clear inhibition zone or a significant reduction in pathogen growth were classified as possessing antifungal potential (Mageshwaran et al., 2022).

### 2.6 Growth parameters assessment

The plant growth-promoting activity of selected rhizospheric isolates was assessed using a germination paper assay under controlled conditions. Surface-sterilized ashwagandha seeds (Vallabh Ashwagandha-1) were treated with bacterial suspensions (108 CFU/mL) and placed on sterile germination paper moistened with sterile distilled water. Samples were incubated in a growth chamber at 25 ± 2 °C with a 12-hour light/dark cycle for 10 days. Control samples were prepared identically but without bacterial inoculation. All experiments were conducted in triplicate. After incubation, growth parameters, including germination percentage, root length, shoot length, fresh weight, and vigor index, were recorded. The Seedling Vigor Index was calculated as SVI = (Root length + Shoot length) × Germination percentage)/100 (Abdul-Baki and Anderson, 1973). Similarly, seeds of the tomato cultivar Gujarat Anand Tomato-5 were utilized to evaluate plant growth–promoting activity under experimental conditions.

### 2.7 Evaluation of *Bacillus subtilis* DMA1 for wilt management in pot trial

Preliminary screening using seed germination and dual-culture assays identified *Bacillus subtilis* DMA1 as having potential antagonistic and growth-promoting activity evaluated under pot conditions. The biocontrol efficacy of DMA1 against *Fusarium solani* was assessed through three treatments: Control, *Fusarium solani* inoculation alone, and co-inoculation of both *Bacillus subtilis* DMA1 and *Fusarium solani*. Growth parameters, including root length, shoot length, root girth, total plant biomass, and disease incidence, were recorded.

### 2.8 Statistical analysis

All analyses were performed using R software (Horton and Kleinman, 2015). Microbial community clustering at the phylum and genus levels was conducted using relative abundance data with the Microeco R package (Liu et al., 2021). Alpha diversity indices, including Good's coverage, observed species, Shannon, and Simpson, were calculated using Phyloseq and Microeco. Beta diversity was evaluated with the Bray–Curtis dissimilarity index and visualized using Principal Coordinate Analysis (PCoA) and hierarchical clustering. Differences in community composition were determined by PERMANOVA. Functional annotation of the shotgun metagenomic reads was performed by aligning high-quality sequences against the KEGG Orthology (KO) database using DIAMOND tool (Bagcl et al., 2021) (blastx mode) with an e-value cutoff of 1e−5. KO assignments were based on the best-hit criterion and classified into KEGG Level 1 and Level 2 pathways. To account for variation in sequencing depth, KO counts were normalized using relative abundance scaling by dividing individual KO counts by the total annotated reads per sample. Statistical comparisons of KEGG Level 2 pathway abundances among the three groups (GN, GV, and RN) were conducted using the Kruskal–Wallis test. Statistical significance was considered at p < 0.05. Functional profiles were visualized using the R statistical environment. Correlation analysis matrices, Mantel tests, and Pearson correlation analyses were performed on soil chemical properties and the relative abundance of dominant bacterial phyla, using the vegan package for the Mantel test and base R functions for the Pearson correlation analyses. Antifungal and growth-promotion activities were evaluated using one-way ANOVA followed by Tukey's HSD test (*P* ≤ 0.05). All wet lab experiments were conducted in triplicate, and results are presented as mean ± standard deviation (SD).

## 3 Results

### 3.1 Metagenomic analysis and taxonomic assignment of soil samples

**Figure 1:**
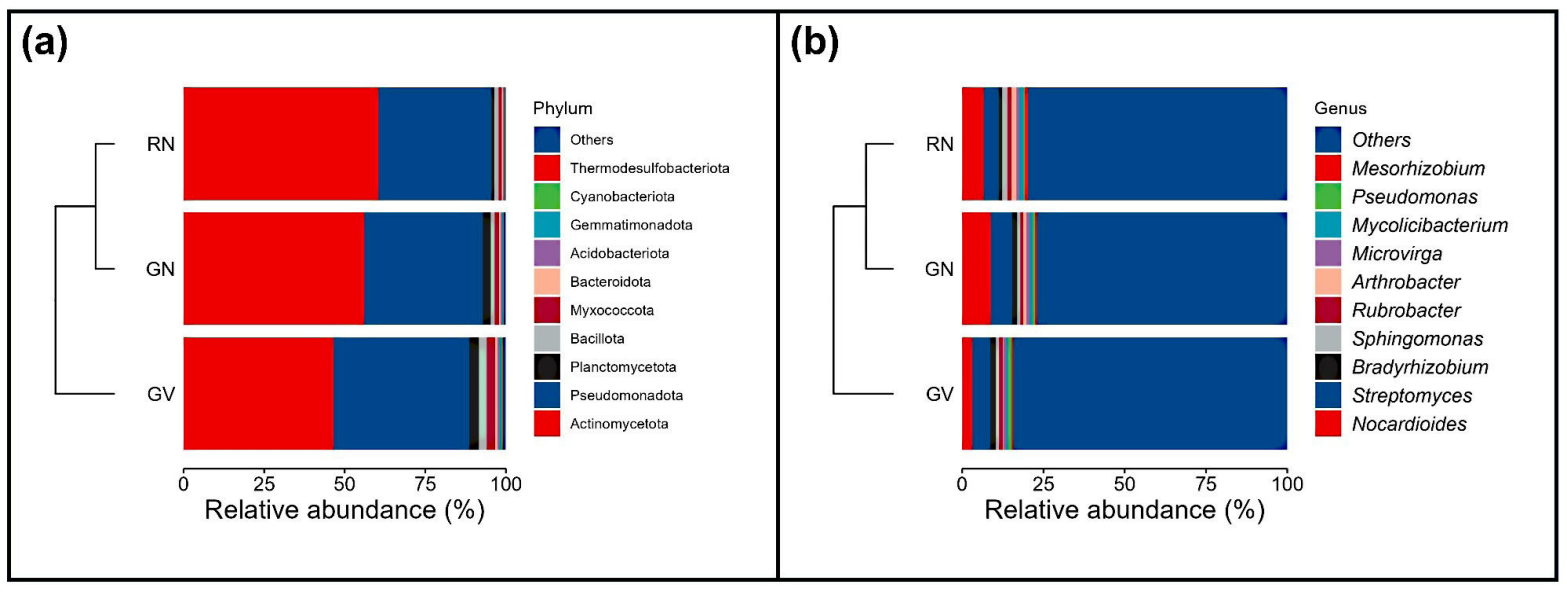
Hierarchical clustering-based relative abundance of dominant bacterial taxa in the rhizosphere of landrace ‘Nagori Ashwagandha' (GN and RN) and Vallabh Ashwagandha-1 (GV) across three experimental sites. **(a)** Phylum-level distribution and **(b)** Genus-level distribution.

Shotgun sequencing generated 232.56 million raw reads across all samples, averaging 38.76 million per sample (range: 30.84 to 58.94 million). After quality filtering, 228.48 million high-quality reads remained, averaging 38.08 million per sample (range: 30.14 to 57.60 million). These reads were classified taxonomically using Kraken2 and the GTDB v207 database for bacterial identification. In total, 17.04 million reads were assigned to bacterial taxa, resulting in a diverse community of 9,356 bacterial species in the final dataset. Taxonomic assignment identified distinct microbial community patterns among the three treatment groups. Clustering analysis indicated that the Landrace ‘Nagori' groups (GN and RN) shared more similar bacterial taxa than the Vallabh ashwagandha-1 group (GV) (Figures 1a, b). The dominant bacterial phyla across all samples were Actinomycetota, Pseudomonadota, Planctomycota, Myxococcota, and Bacillota. GN and RN exhibited more similar phyla distributions than GV (Figure 1a). In GN, Actinomycetota (56%), Pseudomonadota (36%), Planctomycota (2.6%), Myxococcota (2%), and Bacillota (1.5%) comprised 98.1% of total abundance. In RN, Actinomycetota (60%), Pseudomonadota (35%), Planctomycota (1%), Bacillota (1.5%), and Myxococcota (1%) accounted for 98.5%. The GV group consisted of Actinomycetota (46%), Pseudomonadota (42%), Planctomycota (3%), Myxococcota (2.5%), and Bacillota (2.5%), totaling 96%. At the genus level, Nocardioides, *Streptomyces*, and *Bradyrhizobium* were the top three genera in all groups (Figure 1b). In GN, Nocardioides (8.8%), Streptomyces (6.5%), and Bradyrhizobium (1.5%) were most abundant. RN was characterized by Nocardioides (6.6%), Streptomyces (4.5%), and Bradyrhizobium (1.0%), while GV showed lower Nocardioides (3.1%) but higher Streptomyces (5.6%) and Bradyrhizobium (1.6%). Overall, the Nagori variety (GN and RN) supported a more Actinomycetota-dominated rhizosphere, whereas GV was relatively enriched in Pseudomonadota, highlighting the influence of host variety on rhizosphere microbial composition.

**Figure 2:**
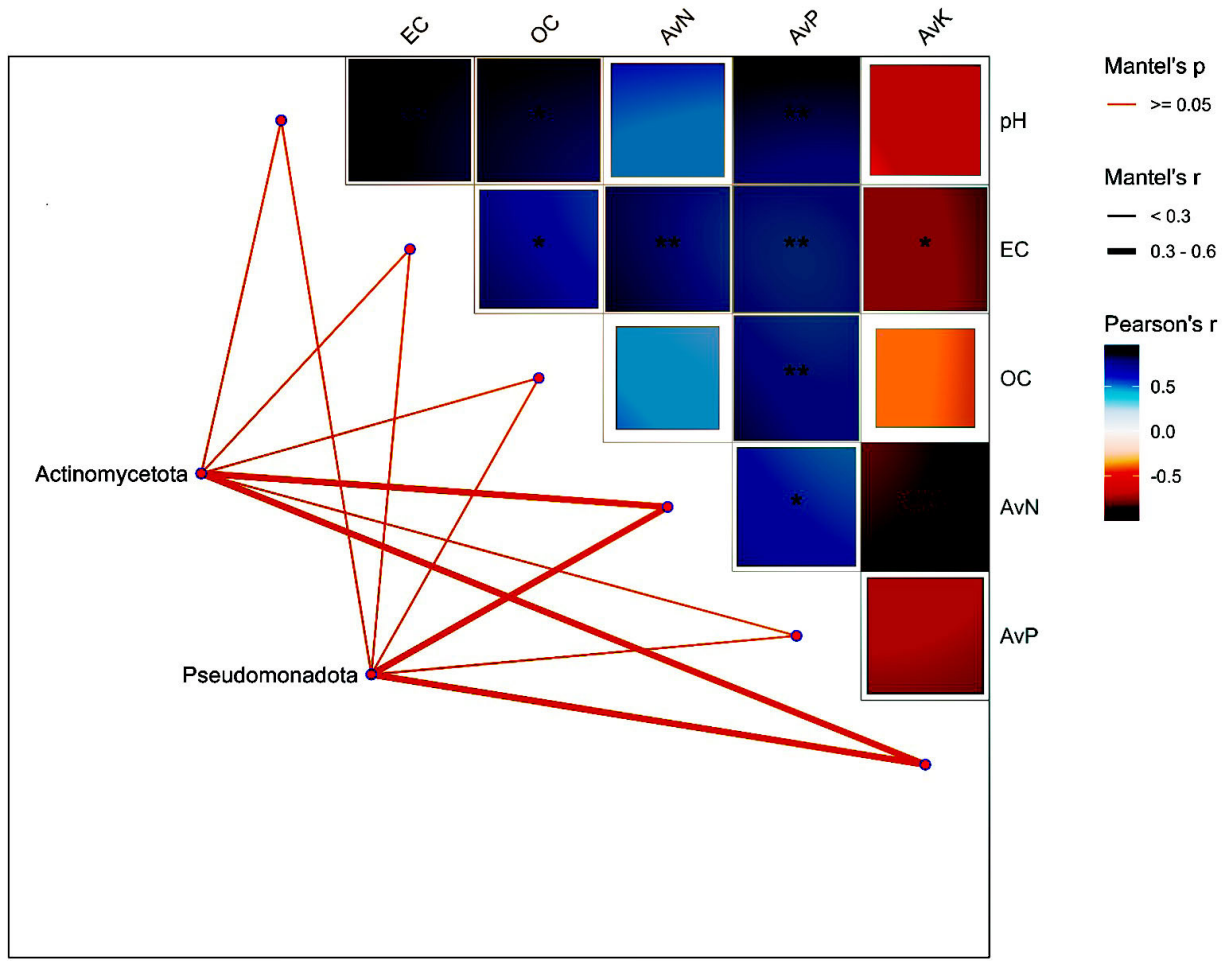
Correlation analysis between dominant bacterial phyla and soil physicochemical properties. Pearson correlation coefficients (r) among soil variables (pH, EC, OC, available N, P, and K) are presented in the upper triangular matrix, with color intensity indicating the strength and direction of the correlation (blue = positive; red = negative). Asterisks indicate significance levels (*p* < 0.05, *p < 0.01, **p < 0.001). Mantel tests were performed to assess the association between bacterial community composition (Actinomycetota and Pseudomonadota) and soil properties. Line thickness represents the strength of Mantel's correlation coefficient (r), and line color indicates statistical significance (*p* ≥ 0.05 as indicated).

### 3.2 Diversity and functional analysis

**Figure 3:**
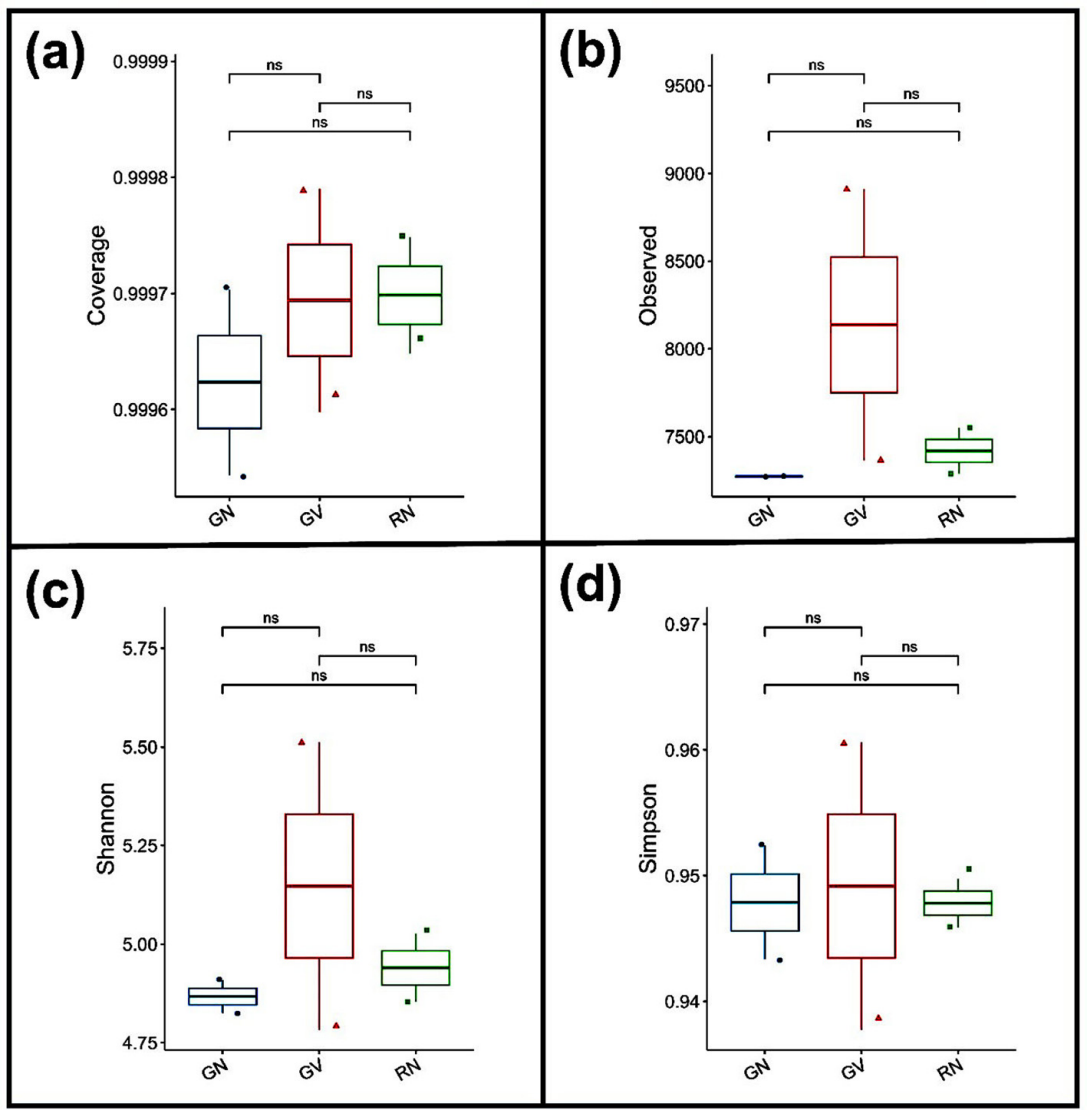
Alpha diversity analysis of rhizosphere bacterial communities associated with GN, GV, and RN. **(a)** Good's coverage, **(b)** observed species richness, **(c)** Shannon diversity index, and **(d)** Simpson diversity index. Boxplots represent median values with interquartile ranges; whiskers indicate minimum and maximum values. Differences among genotypes were evaluated using one-way ANOVA followed by Tukey's HSD test. “ns” indicates non-significant differences (*p* > 0.05).

Alpha diversity metrics showed variation among all groups, but this variation was not statistically significant (*p*<*0.05*) (Figure 3). Good's coverage values were consistently high across all genotypes, at 0.99, indicating that sequencing depth captured most bacterial taxa (Figure 3a). Observed species richness was highest in the GV group, followed by the RN and GN groups (Figure 3b). The Simpson index exhibited a similar pattern, with GV showing the highest value, followed by GN, and RN the lowest (Figure 3d). Overall, GN and RN groups displayed similar diversity patterns compared to GV (Figures 3a-d). However, alpha-diversity metrics did not differ significantly (p > 0.05).

**Figure 4:**
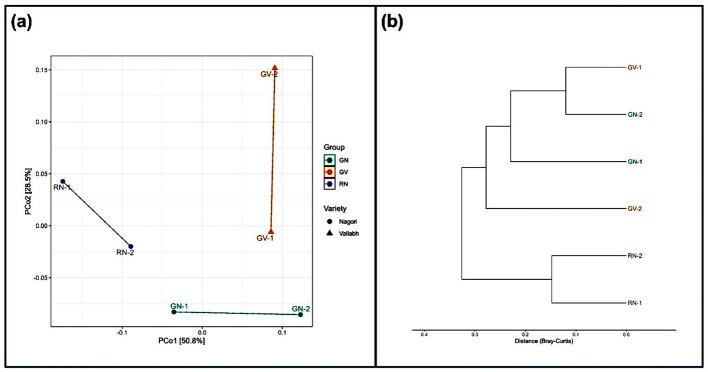
Beta diversity analysis of rhizosphere bacterial communities associated with GN, GV, and RN. **(a)** Principal Coordinates Analysis (PCoA) based on Bray–Curtis dissimilarity showing clustering patterns among samples. **(b)** Hierarchical clustering dendrogram constructed using Bray–Curtis distance, illustrating similarity relationships among samples.

Beta diversity was evaluated using the Bray-Curtis distance index and visualized through PCoA and dendrogram plots. The GN and RN groups clustered closely in ordination space relative to the GV group. The first two principal coordinates explained 50.8% and 28.5% of the variation, together accounting for 79.3% of the variability in bacterial community composition (Figure 4a). Hierarchical clustering further grouped GN and RN within the same clade, while GV formed a separate cluster (Figure 4b). Although partial clustering trends were observed, PERMANOVA analysis indicated that genotype did not significantly affect overall community composition (*p* > 0.05). These findings suggest that genotype may influence community structure; however, the differences were not statistically significant under the tested conditions.

**Figure 5:**
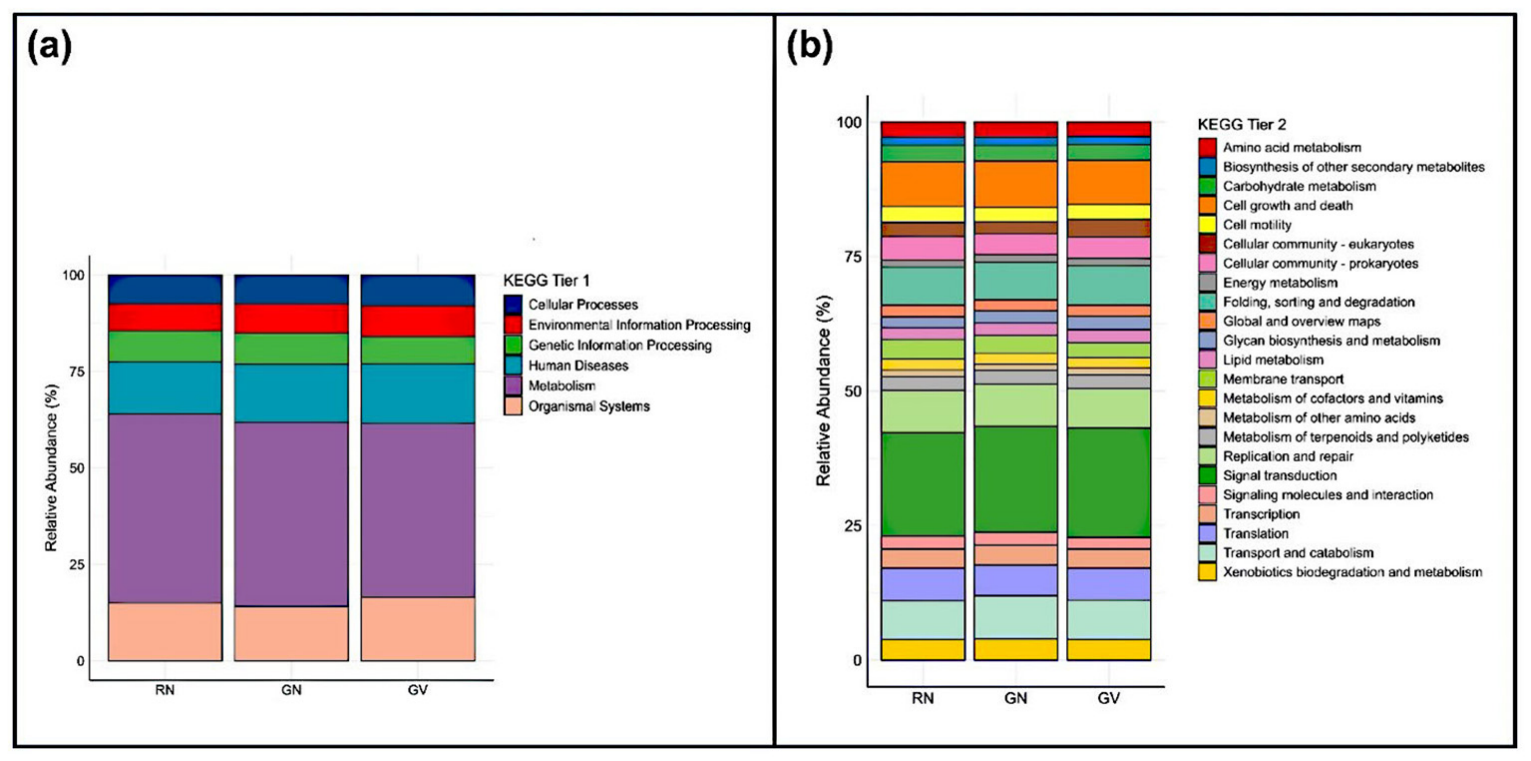
KEGG-based functional profiling of rhizosphere bacterial communities associated with GN, GV, and RN. Relative abundance (%) of predicted functional pathways is shown at **(a)** KEGG Tier 1 categories and **(b)** KEGG Tier 2 categories. Functional annotations were assigned using KEGG database classifications, and values represent the proportional distribution of predicted metabolic pathways across genotypes.

Functional profiling based on Kyoto Encyclopedia of Genes and Genomes (KEGG) annotation showed that Tier-1 categories namely Metabolism, Organismal Systems, and Human Diseases were predominant across all genotypes; however, these assignments primarily represent conserved, homologous pathway modules rather than pathways directly related to disease processes (Figure 5a). Similarly, Tier 2 modules such as signal transduction, biosynthesis of secondary metabolites, cell growth and death, and folding, sorting, and degradation were prevalent in all groups (Figure 5b). No significant variations were observed in Tier 1 and Tier 2 KEGG modules, indicating that microbial functional capabilities are conserved across genotypes.

### 3.3 Soil chemical properties and their correlations with the bacterial taxa

Soil pH ranged from 7.8 to 8.0 across genotypes, indicating alkaline conditions across treatments. Electrical conductivity (EC) varied between 0.14 and 0.26 dS m^−1^. Organic carbon (OC) content was highest in GN (0.53%), followed by RN (0.30%) and GV (0.24%). Available nitrogen (AvN) was highest in GN (244.6 kg ha^−1^), followed by GV (219.5 kg ha^−1^) and RN (175.65 kg ha^−1^). Available phosphorus (AvP) was also highest in GN (33.2 kg ha^−1^), with GV (22.7 kg ha^−1^) and RN (20.3 kg ha^−1^) showing lower levels. Conversely, available potassium (AvK) was highest in RN (566.5 kg ha^−1^), followed by GV (404 kg ha^−1^) and GN (326.2 kg ha^−1^). The physicochemical properties of soil at the two experimental sites are summarized in Table 2. The Mantel test was used to evaluate correlations between soil chemical properties (pH, EC, OC, AvN, AvP, and AvK) and the relative abundance of two bacterial phyla (Actinomycetota and Pseudomonadota). This analysis revealed moderate correlation coefficients (*r* = 0.3–0.6) between soil nutrients (AvK and AvN) and the relative abundance of Actinomycetota and Pseudomonadota. Pearson correlation analysis indicated a negative trend between AvK and other soil parameters, whereas pH, EC, OC, AvN, and AvP exhibited positive intercorrelations (Figure 2).

**Table 2:** The chemical properties analysis of soil samples across the groups.

**Group**	**Soil texture**	**pH**	**EC (dS m^−1^)**	**OC (%)**	**AvN (kg ha^−1^)**	**AvP (kg ha^−1^)**	**AvK (kg ha^−1^)**
GN	clay loam type	8.0 ± 0.10	0.24 ± 0.0045	0.54 ± 0.01	245.3 ± 0.75	34.25 ± 1.05	326.8 ± 0.60
GV	clay loam type	7.7 ± 0.025	0.18 ± 0.005	0.24 ± 0.0025	219.5 ± 1.20	22.7 ± 0.55	404. ± 3.25
RN	sandy loam type	7.7 ± 0.05	0.14 ± 0.01	0.30 ± 0.01	175.6 ± 2.4	20.3 ± 1.2	566.5 ± 1.8

All the values are presented in the mean ± standard error (n=2).

### 3.4 Identification of native bacterial isolates from Ashwagandha rhizosphere

The seventeen isolates were obtained from culturomics at two rhizospheric locations listed in Supplementary Table 1, along with their NCBI accession numbers

*Bacillus* species were represented in metagenomic profiles from both locations and included multiple strains, including *Bacillus subtilis* (ASN1, DMA1, DMA5, AF14, ASN16), *Bacillus altitudinus* (DMA2), and *Bacillus tequilensis* (CM6). Within the genus *Pseudomonas, Pseudomonas synxantha* IS5 and *Pseudomonas oryzihabitans* A15 were identified. Members of Actinobacteria, particularly *Nocardiopsis*, were detected in both sequencing-based analyses and culturable fractions, represented by *Nocardiopsis salina* AN10 and *Nocardiopsis dassonvillei* A5. The identification of *Achromobacter xylosoxidans* A26*, Achromobacter xylosoxidans* AN2 *Mesobacillus foraminis* ASN10*, Brevibacillus nitrificans Ngy2, Priestia aryabhattai* ASN8 and *Leclercia tamurae* PL2, in both metagenomic data and cultured isolates, highlight the overlap between culture-dependent and culture-independent approaches. Pure cultures of *Bacillus subtilis* DMA1 (NAIMCC-B-04593) were deposited at the National Agriculturally Important Microbial Culture Collection (NAIMCC-WDCM60), Mau, Uttar Pradesh, India.

### 3.5 *In vitro* antifungal and plant growth promotion activity against the *Fusarium* wilt pathogen

The antagonistic activity and plant growth-promoting potential of 17 microbial treatments were evaluated *against Fusarium solani* AF2 (PQ066037) isolated from ICAR-DMAPR Boriavi Anand, Gujarat (Shreedevasena et al., 2025). *Bacillus subtilis* DMA1 exhibited the highest mycelial inhibition (64%), with germination percentage (98%), root length (2.1 cm), and shoot length (1.3 cm), seed vigor index (333.2), and maximum fresh biomass (1.12 g), demonstrating both antagonistic and growth-promoting efficacy. *Pseudomonas synxantha* IS5 with 60% mycelial inhibition, high germination (83%), root length 2.0 cm; shoot length 1.2 cm, seed vigor index (265.6), and increased fresh biomass (1.02 g). Similarly, *Bacillus tequilensis* CM6 showed antagonistic activity (58%), with 78% germination rate, seed vigor (210.6), and fresh biomass (0.98 g). *Brevibacillus nitrificans Ngy* 2, *Leclercia tamurae* PL2 and *Mesobacillus foraminis* ASN10 showed 23–28% mycelial inhibition with reduced growth-promoting activity (Supplementary Figure 1). Tomato seeds treated with *Bacillus subtilis* strain DMA1 showed significant growth improvements. Plant height and root length increased by 1.4 and 1.5 times, respectively, compared to untreated seeds. Fresh shoot biomass was 1.4 times higher, while fresh root weight was slightly higher than that of controls (Supplementary Figure 2).

### 3.6 Evaluation of *Bacillus subtilis* DMA1 for Wilt Management under Pot Trial

**Figure 6:**
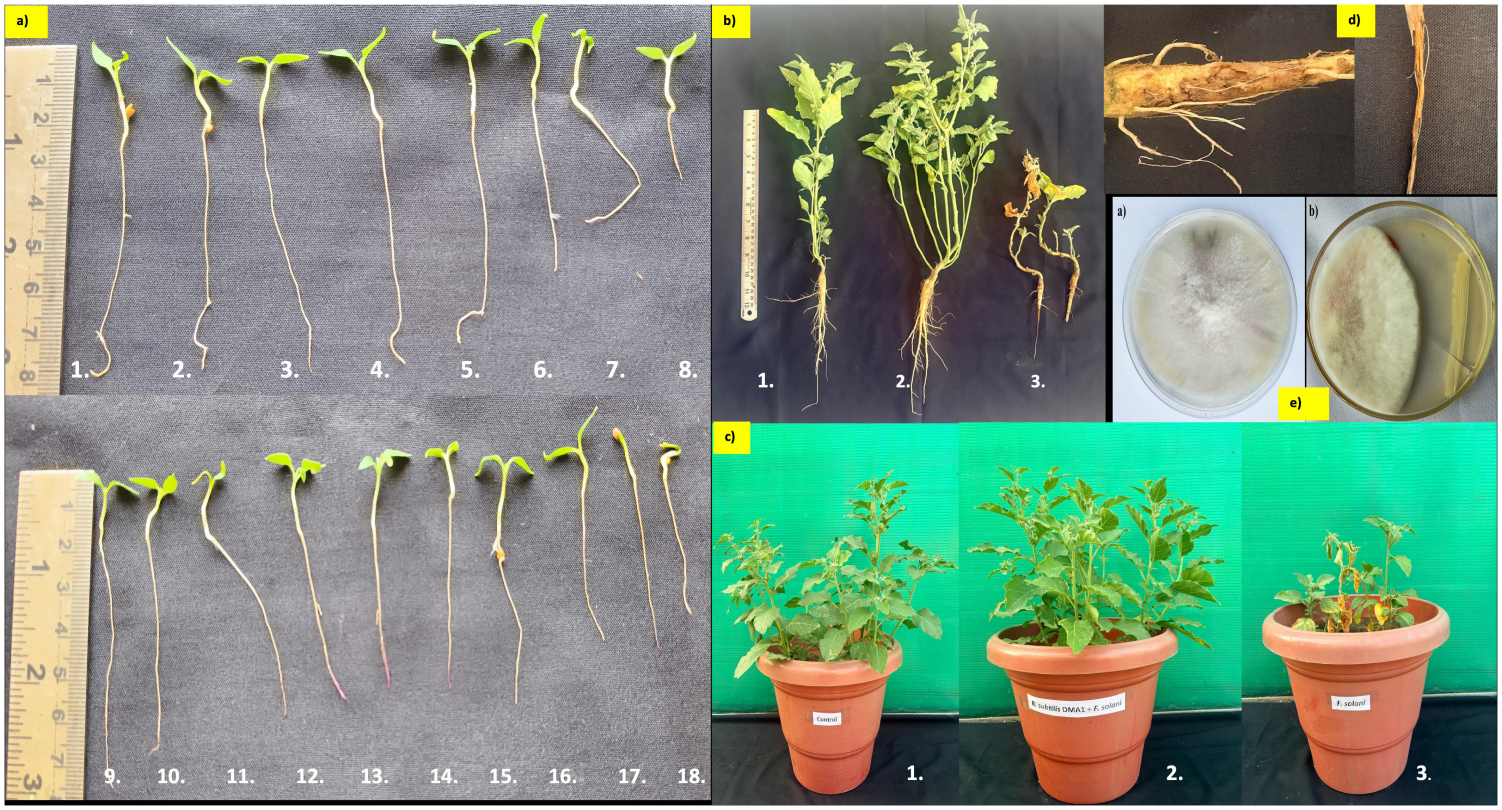
Evaluation of bacterial isolates for growth promotion and biocontrol activity against Fusarium solani. **(a)** Roll paper towel germination assay showing the effects of seventeen bacterial isolates on seedling growth (1. Bacillus subtilis DMA1, 2. Pseudomonas synxantha IS5, 3. Bacillus tequilensis CM6, 4. Bacillus altitudinis DMA2, 5. Bacillus subtilis ASN16, 6. Bacillus subtilis AF 14, 7. Pseudomonas oryzihabitans A15, 8.Control, 9. Nocardiopsis salina AN10, 10.Nocardiopsis dassonvillei A510, 11. Bacillus subtilis DMA5, 12. Bacillus subtilis ASN1, 13. Priestia aryabhattai ASN8, 14. Mesobacillus foraminis ASN10, 15. Leclercia tamurae PL2, 16. Achromobacter xylosoxidans AN2, 17. Achromobacter xylosoxidans A26, 18. Brevibacillus nitrificans Ngy 2) **(b)** and **(c)** Pot experiment demonstrating the effect of Bacillus subtilis DMA1 on plant growth and disease suppression under pathogen challenge: (1) Control, (2) Fusarium solani + Bacillus subtilis DMA1 (co-inoculation), and (3) Fusarium solani alone. **(d)** Root infection symptoms caused by Fusarium solani, showing characteristic discoloration and tissue necrosis. **(e)** Dual culture assay **(a)** Fusarium solani control plate and **(b)** Mycelial inhibition of Bacillus subtilis DMA1.

The wilt incidence of ashwagandha was 67% under field conditions, and infected plants showed yellowing leaves and wilting symptoms accompanied by root rot*Fusarium solani* alone significantly reduced root length (7.4 cm), shoot length (19.1 cm), root girth (7.23 mm), and total biomass (19.4 g) compared to the control (15.3 cm, 28.4 cm, 9.25 mm, and 28.3 g, respectively), with the highest disease incidence of 80%. Co-inoculation with *F. solani* and *Bacillus subtilis* DMA 1 significantly increased root length (20.1 cm), shoot length (39.5 cm), root girth (14.9 mm), and total biomass (51.1 g) compared to both control and pathogen-alone treatments (Figure 6, Supplementary Figure 3). Disease incidence was reduced by 70% in *Bacillus subtilis* DMA1 treatment.

## 4 Discussion

This study examined the effects of Ashwagandha genotypes on soil chemical properties, microbial community structure, diversity, and functional profiles across two agroecological locations. A comparative analysis of the rhizomicrobiome of Nagori and Vallabh ashwagandha-1 was conducted to elucidate the combined effects of plant genotype and geography. Metagenomic sequencing revealed a highly diverse bacterial community, with approximately 9,000 bacterial species identified across three groups. Community composition analysis indicated greater similarity between GN and RN samples (Nagori) than between GV (Vallabh) and GN/RN samples, suggesting that host plant genotype is a primary determinant of rhizosphere microbial structure, even across distinct geographic locations. This genotype-driven clustering is consistent with previous research demonstrating strong host control over rhizosphere microbiome assembly (Singh et al., 2022; Zhang et al., 2022; Zheng et al., 2023; Steinberger et al., 2024; Sutherland et al., 2022). Actinomycetota were the dominant bacterial phylum in Nagori (GN and RN), whereas the Vallabh variety (GV) showed a higher relative abundance of Pseudomonadota. The predominance of Actinomycetota in the Nagori rhizosphere suggests a genotype-associated core microbiome adapted to nutrient-poor, semi-arid soils. Actinomycetota are recognized for producing dormant spores and osmoprotective compounds, which enhance resilience under low-moisture and nutrient-limited conditions (Delgado-Baquerizo et al., 2018; Naylor et al., 2017). At the genus level, *Nocardioides, Streptomyces*, and *Bradyrhizobium* were dominant and are known for their plant growth-promoting functions, including nitrogen fixation, phytohormone production, and antimicrobial activity (Hnini and Aurag, 2024). The reduced abundance of these genera in the GV group indicates weaker maintenance of functionally important taxa, potentially due to genotype-specific root exudation patterns or microbe–plant compatibility shaped by agroecological and edaphic factors. Alpha diversity indices in the GV group consistently showed higher richness and evenness, as indicated by observed species counts, Shannon's index, and Simpson's index, and increased diversity was associated with lower nutrient availability, suggesting that nutrient richness alone does not determine microbial diversity. Nutrient-rich environments may promote dominance by fast-growing taxa, reducing community evenness (Wang et al., 2020; Zhu et al., 2022). Although the difference was not statistically significant, a similar observation was also reported by Alawiye and Babalola (2021) in the sunflower rhizosphere microbiome. GN and GV were cultivated under similar environmental conditions; the observed differences in diversity likely reflect genotype-driven effects, potentially mediated by variations in root exudate composition and secondary metabolite profiles that influence microbial recruitment (Anderson et al., 2024; Han et al., 2025). Beta-diversity analyses further supported these findings, with partial clustering of GN and RN together, and GV forming a distinct group, reinforcing the influence of plant genotype on rhizosphere microbial assemblages. Functional profiling using KEGG pathway analysis revealed that core microbial functions related to metabolism, signal transduction, and cell growth and death were conserved across all groups. This functional redundancy indicates that, despite taxonomic shifts, essential ecological processes remain stable within the rhizosphere. Such redundancy is considered essential for maintaining ecosystem resilience and functional stability under environmental stress, a pattern frequently observed in soil microbiomes (Chen et al., 2022).

Variations in soil chemical properties among the three groups (GN, GV, and RN) reflect the combined effects of site-specific edaphic conditions and host genotypes. The Gujarat-grown Nagori (GN) group exhibited higher soil pH, electrical conductivity, organic carbon, and macronutrient availability, particularly nitrogen and phosphorus, indicating a nutrient-enriched rhizosphere. Previous studies have demonstrated that elevated organic carbon and available nitrogen are key drivers of microbial activity and nutrient turnover, supporting a more functionally active microbial community (Prommer et al., 2020; Chauhan et al., 2023; Lv et al., 2024). The Nagori group in Rajasthan (RN) displayed the highest available potassium levels but lower concentrations of other soil nutrients. This pattern likely reflects the mineralogical properties of Rajasthan soils, which are often enriched in potassium minerals, rather than being solely attributable to varietal effects (Sharma et al., 2023). Such edaphic constraints limit organic matter accumulation and nutrient availability, thereby shaping distinct rhizosphere conditions. Gujarat Vallabh (GV) group exhibited intermediate soil chemical parameters, with lower organic carbon content than GN. Although both GN and GV were cultivated under the same conditions, these differences suggest a potential genotype influence on rhizosphere nutrient dynamics within the same geographic setting (Steinberger et al., 2024). The results for GN indicate enhanced organic carbon and nitrogen availability, favoring greater microbial activity and nutrient mineralization, whereas the elevated potassium levels in RN soils primarily reflect intrinsic soil mineralogy. Collectively, these findings underscore the combined influence of plant genotype and regional soil characteristics on the structuring of rhizosphere nutrient environments. Mantel test analysis revealed moderate positive correlations between the dominant bacterial phyla (Actinomycetota and Pseudomonadota) and the availability of nitrogen and potassium. These trends align with previous reports indicating that soil nutrient availability influences microbial community composition, often in a genotype-dependent manner (Sharma et al., 2023). The preferential enrichment of Actinomycetota in potassium-rich, nutrient-limited soils may confer a selective advantage by enhancing nutrient mobilization and stress tolerance in arid environments (Delgado-Baquerizo et al., 2018). Additionally, available potassium exhibited a negative correlation with most other soil chemical properties, suggesting complex nutrient trade-offs within the rhizosphere. These interactions may indirectly influence microbial community dynamics by altering nutrient accessibility and competitive interactions among microbial taxa (Steinberger et al., 2024).

A consistent set of dominant bacterial phyla was identified across both geographic locations, indicating a core Ashwagandha-associated microbiome. Actinomycetota were consistently detected at both sites, although their relative abundance varied with environmental conditions. Few studies have examined both culturable and unculturable microbial diversity in Ashwagandha; however, these findings align with rhizosphere microbiome studies in other crops, such as citrus, where similar phyla were observed across locations and seasons (Lima et al., 2024). Traditional culture-based methods capture only a small fraction of total microbial diversity (0.1–10%), underscoring the importance of metagenomic approaches for comprehensive microbiome characterization (D'Onofrio et al., 2010; Goulart et al., 2019).

A comparative analysis of culture-independent metagenomic profiling and culture-dependent isolation demonstrated strong concordance between the dominant bacterial taxa in the Ashwagandha rhizosphere and the native bacterial strains isolated from it. Shotgun metagenomic sequencing and culturomics analysis identified *Bacillaceae, Pseudomonadaceae, Nocardiopsaceae*, and *Alcaligenaceae* as the predominant families. Functional annotation of metagenomic reads using the KEGG database revealed enrichment of pathways related to secondary metabolite biosynthesis, nutrient cycling, and stress response. The combined use of metagenomics and culturomics enabled the identification of dominant, functionally relevant bacterial taxa in the Ashwagandha rhizosphere, thereby supporting the selection of promising native isolates for biocontrol and plant growth promotion This functional convergence indicates that culturable members are key contributors to the rhizosphere microbiome's metabolic potential.

The functional significance of the rhizosphere microbiome was further demonstrated by the antifungal activity of bacterial isolates against *Fusarium solani*, the causal agent of Ashwagandha wilt. Numerous studies have established the biocontrol efficacy of *Bacillus subtilis* and *Pseudomonas fluorescens* against soil-borne pathogens, including *Fusarium* species, through mechanisms such as antibiosis, competition, and induction of systemic resistance (Gowtham et al., 2016; Khan et al., 2018; Duan et al., 2023; Lu et al., 2024). The functional profiling further indicated the presence of genes associated with terpenoid and polyketide metabolism in the rhizosphere metagenomes, which are putatively linked to microbial secondary metabolite production and may contribute to plant defense–related interactions in the rhizosphere *B. subtilis* DMA1 exhibited strong *in vitro* antagonistic activity (64% inhibition) against *F. solani* reduces disease incidence by 70% in pot trials with increased growth-promoting activity, highlighting its potential as a beneficial rhizosphere inhabitant that contributes to disease suppression. Although reports on the biological control of Ashwagandha wilt are limited, these results, together with previous studies on stress mitigation and growth promotion by *B. subtilis* strains (Patel et al., 2023) underscore the importance of indigenous rhizosphere microbes in promoting plant health. Collectively, these findings suggest that genotype-driven recruitment of beneficial microbial consortia may enhance stress tolerance and disease resistance in Ashwagandha. The study demonstrates that Ashwagandha genotype strongly influences soil chemical properties, rhizosphere microbial composition, and functional potential. The indigenous Nagori genotype consistently supports a microbiome enriched in stress-resilient Actinomycetota across contrasting environments, reinforcing its adaptation to semi-arid agroecological conditions. These results emphasize the importance of integrating plant genotype, soil chemistry, and microbiome interactions in future strategies for sustainable cultivation and microbiome-assisted breeding of medicinal plants.

## 5 Conclusion

The rhizospheric microbial communities of two Ashwagandha genotypes, Nagori and Vallabh-1, were analyzed at two distinct agroecological sites. The results indicate that plant genotype significantly affects both soil chemical properties and rhizomicrobiome composition. The Nagori genotype in Rajasthan (RN) and Gujarat (GN) consistently exhibited Actinomycetota-dominated microbiomes, demonstrating that genotype-specific microbial associations persist across locations. Both metagenomic and laboratory analyses showed that Nagori and Vallabh Ashwagandha-1 (VA-1) predominantly recruited *Bacillus* and *Pseudomonas*, taxa recognized for their antifungal and biocontrol properties. These findings clarify genotype-specific recruitment patterns and suggest the potential to incorporate beneficial microbial communities into sustainable Ashwagandha production. Overall, the study highlights that host genotype-microbiome interactions can support the development of climate-resilient medicinal crops through microbiome-assisted breeding, especially in nutrient-poor, semi-arid soils, thereby improving stress resilience, nutrient use efficiency, and bioactive compound accumulation.

## Data Availability

The datasets presented in this study can be found in online repositories. The names of the repository/repositories and accession number(s) can be found in the article/Supplementary material.
