# Supplementary material for: Deciphering genotype and geography dependent microbiome composition and its role in disease suppression in Ashwagandha
Source: Front Microbiol. 2026 Mar 20;17:1786817. doi: 10.3389/fmicb.2026.1786817 (PMC13047081; doi:10.3389/fmicb.2026.1786817)
Supplement: Supplementary file 1 [file Table_1.DOCX]

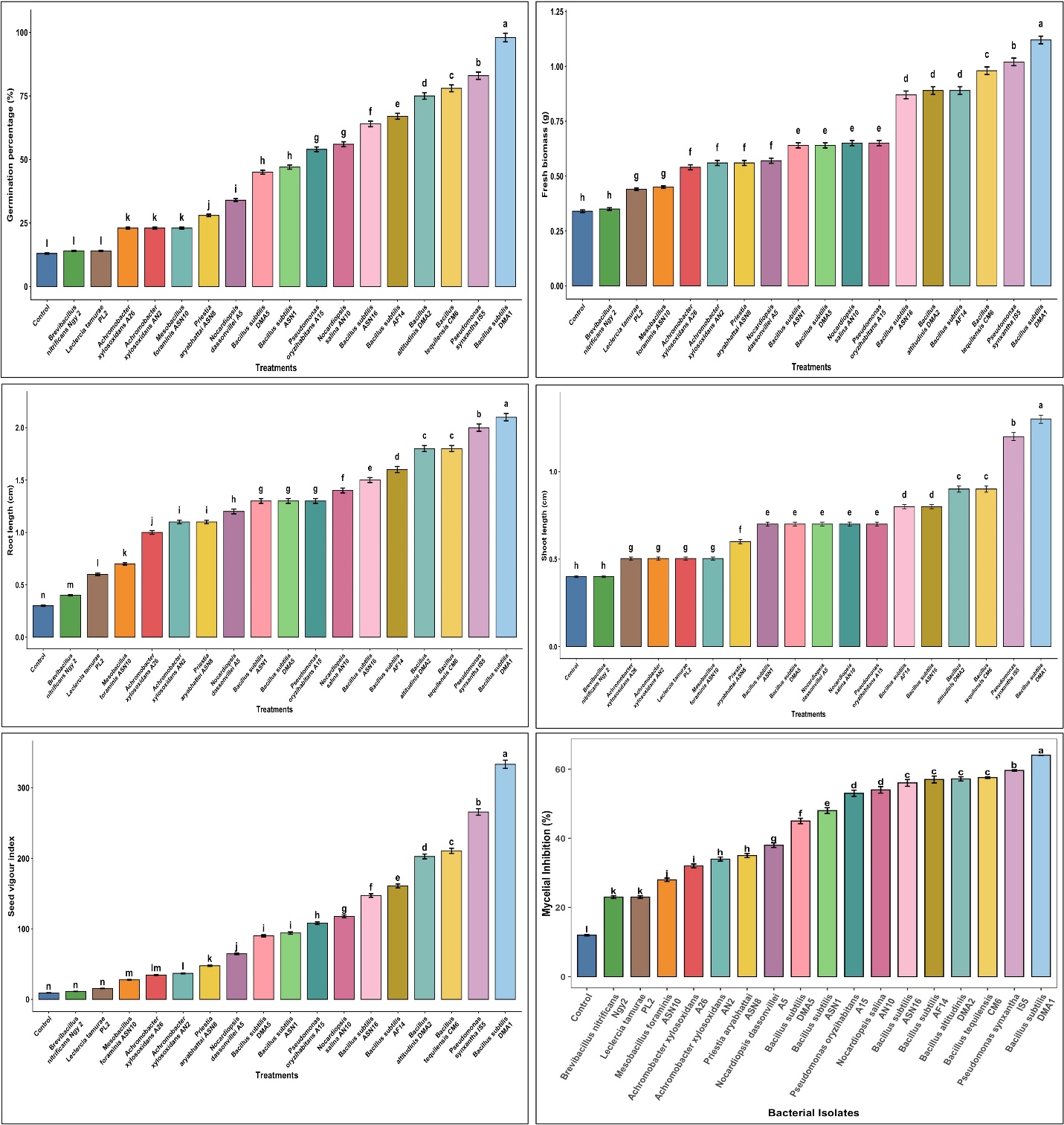


**Supplementary Fig. 1**: Effect of bacterial isolates on seed germination and plant growth parameters. A) Germination percentage, (B) fresh biomass, (C) root length, and (D) shoot length. E) Seed vigour index. F) Percentage inhibition of *Fusarium solani* under dual-culture assay by different bacterial isolates. Data represent mean ± SE of three biological replicates. Different lowercase letters above the bars indicate significant differences among treatments according to Tukey’s HSD test at P ≤ 0.05. One-way ANOVA showed a significant treatment effect for all measured parameters (P < 0.05).


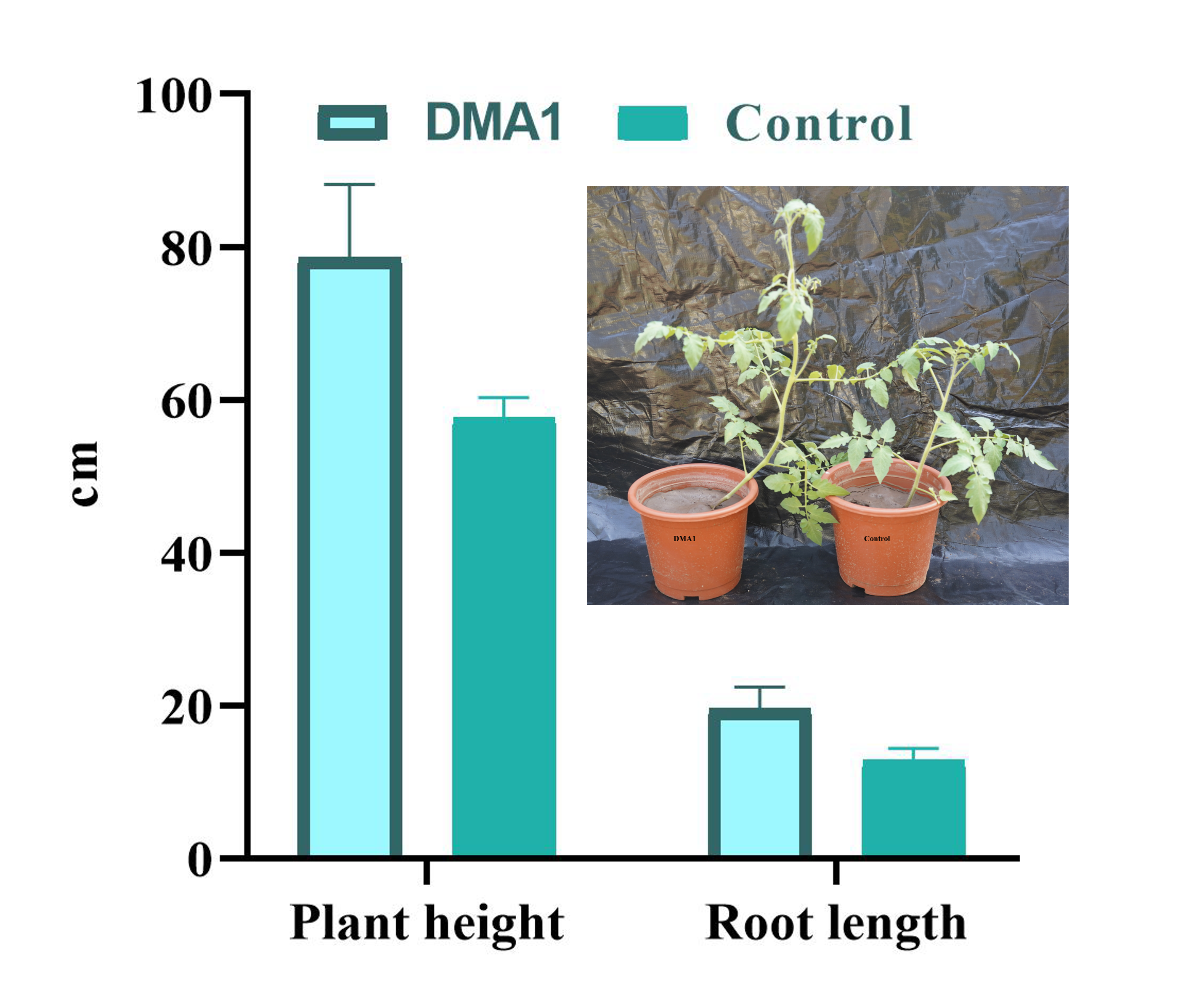


**Supplementary Fig. 2**: Effect of Bacillus subtilis DMA1 on Tomato plant height and root length under pot conditions

*
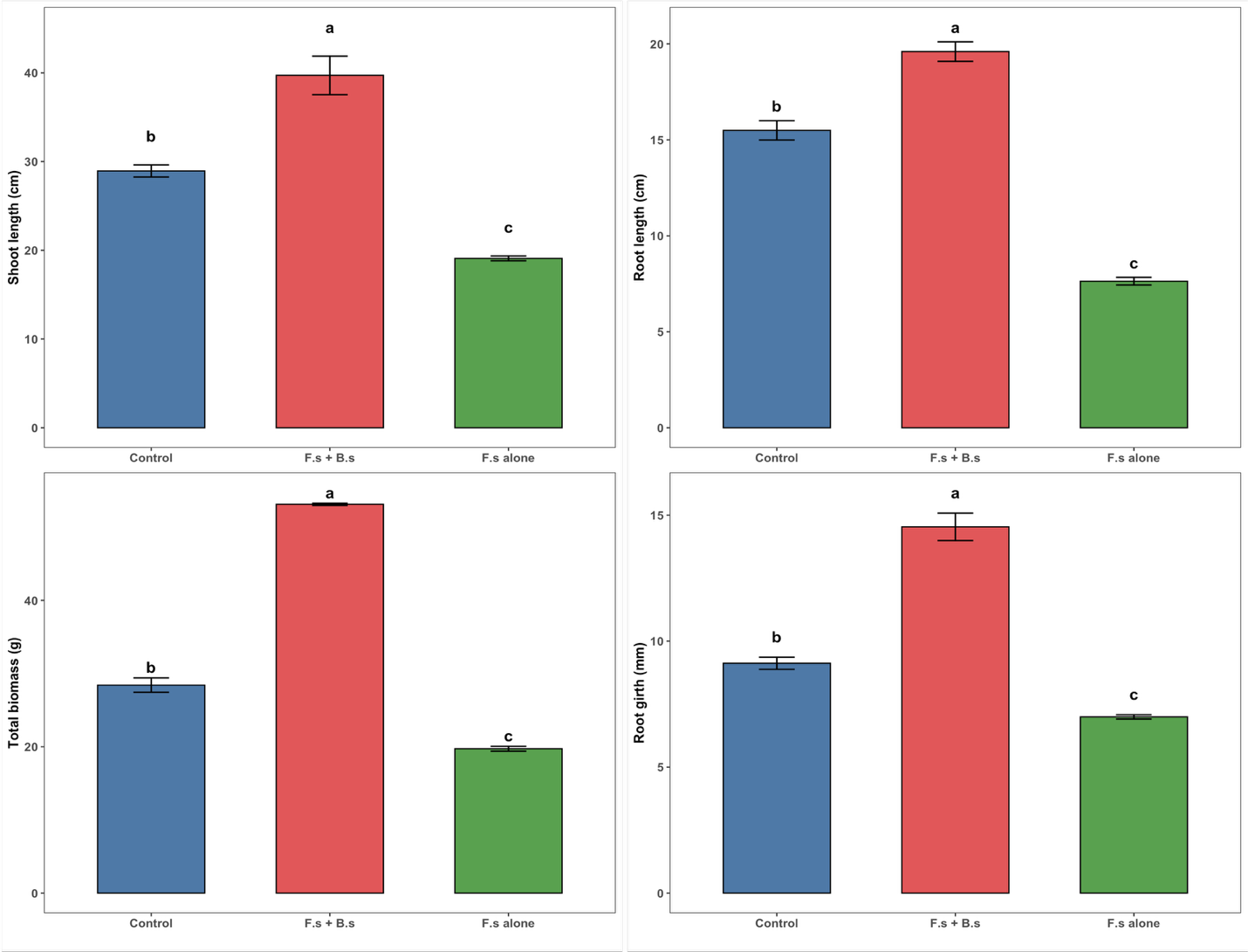
*

### **Supplementary Fig 3. Effect of** Bacillus subtilis DMA1 **on growth parameters under** Fusarium solani. Effect of treatments on (A) shoot length, (B) root length, (C) total biomass, and (D) root girth. Data represent mean ± SE of three biological replicates. Different lowercase letters above bars indicate significant differences among treatments according to Tukey’s HSD test (P ≤ 0.05). One-way ANOVA revealed significant treatment effects for all growth parameters (P < 0.05).
